# Dual pathways construction: a prospective study on how physical activity predicts medical students’ professionalism via reducing academic burnout and enhancing self-efficacy

**DOI:** 10.3389/fpsyg.2026.1796754

**Published:** 2026-05-29

**Authors:** Ming Gao, Qiao Liu, Keqiang Li, Jianye Li

**Affiliations:** 1 Department of Physical Education, Shanxi Medical University, Jinzhong, China; 2 School of Management, Shanxi Medical University, Jinzhong, China; 3 Faculty of Sports Science, Wenzhou Medical University, Wenzhou, China

**Keywords:** academic burnout, medical students, physical activity, professionalism, self-efficacy

## Abstract

**Objective:**

This two-wave prospective study examined whether physical activity was indirectly associated with perceived professionalism among medical students through academic burnout and self-efficacy.

**Methods:**

A total of 625 medical students completed validated measures of physical activity (IPAQ-SF), academic burnout (MBI-SS), general self-efficacy (GSES), and professionalism (PAS) at two time points, 6 months apart. Data were analyzed using structural equation modeling with bootstrapping.

**Results:**

The prospective indirect-effect model showed good fit. T1 physical activity was negatively associated with T2 academic burnout and positively associated with T2 self-efficacy. In turn, T2 burnout and T2 self-efficacy were associated with T2 perceived professionalism. The direct path from T1 physical activity to T2 perceived professionalism was not significant. Significant prospective indirect associations were observed through both burnout and self-efficacy, with no significant difference between the two indirect pathways.

**Conclusion:**

Physical activity was prospectively associated with perceived professionalism through lower burnout and higher self-efficacy. Given the two-wave observational design and the concurrent assessment of mediators and outcome at T2, these findings should be interpreted as prospective indirect associations rather than evidence of causal or full mediation.

## 1 Introduction

The cultivation of physician professionalism represents one of the central objectives of medical education, as the quality of perceived professionalism directly may be related to future professional functioning (Mueller, 2015; Brody and Doukas, 2014). Contemporary perspectives on professionalism extend well beyond the acquisition of technical knowledge and clinical skills. Epstein and Hundert (2002) described perceived professionalism as “the habitual and judicious use of communication, knowledge, technical skills, clinical reasoning, emotions, values, and reflection in daily practice.” This definition highlights the multidimensional nature of professionalism and emphasizes that professionally prepared physicians must possess not only medical expertise, but also emotional resilience, ethical sensitivity, and interpersonal effectiveness in complex clinical environments.

Paradoxically, the educational environment intended to cultivate these competencies may also undermine their development. Medical training is characterized by persistent academic pressure, intensive workload, high-stakes evaluation, and early exposure to illness, suffering, and mortality, all of which place students at increased risk of psychological distress (Dyrbye et al., 2005; George, 2024). One of the most prevalent manifestations of this distress is academic burnout, which typically includes emotional exhaustion, cynicism, and a reduced sense of personal accomplishment (Ramos-Vera et al., 2025; Mabele et al., 2024). Burnout not only impairs students’ psychological well-being but may also compromise empathy, ethical engagement, reflective learning, and sustained motivation, all of which are fundamental components of professionalism-related development. Consequently, identifying modifiable factors capable of reducing psychological depletion while strengthening adaptive functioning has become an important concern in medical education research.

Within this context, physical activity has emerged as a potentially important yet insufficiently explored protective factor. Unlike incidental physical activity, structured exercise is characterized by planned, repetitive, and goal-oriented movement aimed at improving physical fitness and overall functioning (Stavrinou et al., 2025). A substantial body of research has demonstrated the physiological and psychological benefits of regular exercise, including improved cardiometabolic functioning, neuroendocrine regulation, and stress adaptation (Gupta et al., 2024; Tsatsoulis and Fountoulakis, 2006). For medical students, whose future professional responsibilities require sustained cognitive performance, emotional regulation, and physical endurance, exercise may represent more than a leisure activity or health behavior. Rather, it may function as a practical strategy for maintaining psychological resilience and adaptive capacity throughout training (Dunn et al., 2008; Hojat et al., 2003).

Beyond its physiological benefits, physical activity may also be conceptualized as a form of behavioral self-regulation within demanding educational settings. Sustained participation in exercise requires persistence, goal-directed behavior, emotional control, and adaptive coping, all of which overlap with competencies necessary for successful medical training. In this regard, exercise may serve as a behavioral resource that supports psychological adjustment under chronic academic stress. Medical students frequently encounter prolonged workload, emotional fatigue, and continuous performance expectations, conditions that can gradually erode motivation and professional engagement. Regular exercise may therefore help maintain emotional stability, self-regulatory capacity, and long-term engagement with professional learning.

Although previous studies have reported associations between exercise and general well-being or academic performance, the mechanisms through which physical activity may be associated with perceived professionalism through psychological adaptation processes (Li et al., 2025; Wilczyńska and Hejła, 2024). Existing research has often focused on broad mental health outcomes while paying limited attention to how exercise may related to professional attitudes, clinical judgment, and relational professionalism within medical education.

Theoretically, the relationship between exercise and professionalism may operate through intermediary psychological processes that related to how students adapt to the demands of medical training (Elman et al., 2005). In the present study, two potential mediating mechanisms are proposed: academic burnout and self-efficacy. These pathways may reflect two complementary dimensions of psychological adaptation. On the one hand, exercise may reduce maladaptive outcomes associated with chronic stress exposure, particularly burnout, thereby functioning as a protective factor. On the other hand, exercise may strengthen positive psychological resources such as self-efficacy through repeated mastery experiences and perceptions of professionalism. Examining these mechanisms simultaneously may therefore provide a more comprehensive understanding of how exercise associated to professionalism-related development.

From the perspective of conservation of resources theory, burnout develops when individuals experience a chronic imbalance between environmental demands and available psychological resources (Hobfoll and Freedy, 2017). Exercise may counteract this process by facilitating physiological recovery, emotional regulation, and stress adaptation. As a controlled physical stressor, exercise can induce adaptive responses that improve resilience and reduce negative affective states (Koyama, 2014). Through these mechanisms, exercise may interrupt the cycle of chronic stress, emotional exhaustion, and disengagement that often characterizes burnout in medical students. Reduced burnout may subsequently preserve students’ emotional energy and capacity for sustained professional engagement (Nalagamage et al., 2025).

Self-efficacy represents another potentially important mechanism. According to Bandura’s social cognitive theory, self-efficacy refers to individuals’ beliefs regarding their ability to organize and execute behaviors necessary to achieve desired outcomes (Bandura, 1991). Such beliefs are strengthened through mastery experiences, successful performance, and adaptive interpretation of physiological states. Physical activity provides repeated opportunities for observable progress and personal accomplishment, whether through improvements in endurance, strength, or physical skill. These experiences may foster broader beliefs in personal professionalism and resilience that extend into academic and clinical domains. Moreover, positive interpretations of exercise-related physiological arousal may further reinforce efficacy beliefs (Welch et al., 2010). Increased self-efficacy may subsequently promote persistence, adaptive coping, and confidence in professional learning and patient interaction.

Despite the plausibility of these mechanisms, existing evidence remains fragmented. Many studies have focused primarily on isolated bivariate relationships and have rarely integrated exercise, burnout, self-efficacy, and professionalism within a unified longitudinal framework. In addition, much of the existing literature is cross-sectional, limiting the ability to examine temporal associations among these constructs. A longitudinal approach is therefore necessary to clarify whether physical activity prospectively predicts changes in psychological functioning and professionalism over time.

To address these gaps, the present study employed a two-wave longitudinal design to examine a dual-pathway mediation model linking physical activity to professionalism among medical students. Specifically, we proposed that physical activity associated to professionalism through two complementary mechanisms: by reducing academic burnout and by enhancing self-efficacy. In doing so, this study extends the concept of integrating physical activity and medicine beyond clinical health promotion and positions exercise as a potentially important component of professionalism-related development within medical education.

Based on the theoretical and empirical literature reviewed above, the following hypotheses were proposed. H1: Physical activity at T1 will be negatively associated with academic burnout at T2 after controlling for baseline burnout. H2: Physical activity at T1 will be positively associated with self-efficacy at T2 after controlling for baseline self-efficacy. H3: Academic burnout at T2 will be negatively associated with professionalism at T2 after controlling for baseline professionalism. H4: Self-efficacy at T2 will be positively associated with professionalism at T2 after controlling for baseline professionalism. H5: Academic burnout and self-efficacy will show prospective indirect associations linking T1 physical activity with T2 perceived professionalism. H6: The relative strength of the two indirect pathways will be explored to determine whether the relationship between exercise and professionalism is more strongly associated with reduced burnout or enhanced self-efficacy.

To test these hypotheses, a prospective longitudinal design with two measurement waves separated by 6 months was adopted. Physical activity was assessed using the International Physical Activity Questionnaire–Short Form, academic burnout was measured using the Maslach Burnout Inventory–Student Survey, self-efficacy was assessed using the General Self-Efficacy Scale, and professionalism was evaluated using the professionalism Scale for Medical Students. All constructs were measured at both T1 and T2. Baseline levels of mediators and outcome variables were statistically controlled to strengthen temporal interpretation. Structural equation modeling was employed to examine the proposed parallel mediation model while accounting for measurement error. By testing the relative contribution of burnout and self-efficacy simultaneously, this study aims to clarify the psychological mechanisms through which physical activity may support professionalism-related development in medical education.

## 2 Materials and methods

### 2.1 Participants

A prospective longitudinal study was conducted among medical students recruited from three medical universities in China. To improve sample diversity and representativeness, a multi-stage cluster sampling strategy was adopted. First, three medical universities located in different geographic regions of China (eastern and central regions) were selected. Within each participating institution, graduating classes were randomly selected as sampling clusters, and all students within the selected classes were invited to participate.

Across the three universities, a total of 700 students were initially approached, including 238 from Wenzhou Medical University A, 227 from Shanxi Medical University, and 235 from Changzhi Medical University. The inclusion criteria were: (1) full-time enrollment in a clinical medicine program, (2) voluntary participation, and (3) provision of informed consent. Participants were excluded if they were currently on leave of absence, failed to complete key study measures, provided invalid or patterned responses, or reported physical conditions that substantially restricted routine physical activity participation. The latter criterion was applied to improve the comparability and interpretability of habitual physical activity assessment using the IPAQ-SF.

At the first measurement point (T1), 658 students completed the survey, yielding a response rate of 94.0%. During data screening, 18 responses were excluded because of excessive missing data or invalid response patterns, and 15 participants were excluded due to self-reported physical conditions that substantially limited physical activity participation. Six months later, follow-up data were collected at Time 2 (T2). Of the eligible T1 participants, 625 completed the second-wave assessment, resulting in a longitudinal retention rate of 95.0% and a final analytic sample of 625 medical students.

The final sample size exceeded the minimum sample size of 200 commonly recommended for structural equation modeling (SEM) and provided sufficient statistical power (0.95), as estimated using G*Power, to detect small-to-moderate effect sizes.

### 2.2 Procedure

This study utilized a two-wave longitudinal panel design with a six-month interval between measurements. The interval was chosen to allow for potential changes in exercise habits and psychological states across an academic semester while minimizing attrition. Data collection occurred online via a secure survey platform (Wenjuanxing). At T1 (beginning of the fall semester), eligible participants received an invitation link containing the study information sheet and consent form. Upon providing electronic informed consent, they completed the baseline questionnaire assessing demographics, physical activity, academic burnout, self-efficacy, and professionalism. At T2 (end of the spring semester), the same participants received a follow-up link to complete an identical questionnaire battery. To enhance retention, three reminder emails were sent, and participants who completed both waves were entered into a lottery for small incentives. All data were anonymized using unique participant codes. The research protocol was approved by the Wenzhou medical university, approve ID was WMU2025066, and all procedures adhered to the Declaration of Helsinki and applicable national research ethics guidelines.

### 2.3 Instruments

#### 2.3.1 Physical activity

Physical activity levels were assessed using the short form of the International Physical Activity Questionnaire (IPAQ-SF), a widely used self-report instrument developed by the International Physical Activity Questionnaire group (Craig et al., 2003). The IPAQ-SF evaluates the frequency (number of days) and duration (minutes per day) of physical activity performed over the previous 7 days across three intensity domains: walking, moderate-intensity activities, and vigorous-intensity activities. Participants reported, for example, “During the last 7 days, on how many days did you walk for at least 10 min at a time?” and “How much time did you usually spend walking on one of those days?”

Scoring followed the official IPAQ-SF guidelines. Metabolic Equivalent of Task (MET) values were assigned to each activity intensity: walking = 3.3 METs, moderate activity = 4.0 METs, and vigorous activity = 8.0 METs. Weekly MET-minutes for each domain were calculated by multiplying the MET value by the minutes of activity per day and then by the number of days per week. These values were summed to derive a continuous score for total weekly physical activity (IPAQ Total MET-min/week). Data cleaning procedures followed established IPAQ guidelines. Reported activity durations exceeding 180 min per day for a single activity category were truncated to reduce the related to of implausible overreporting. Total physical activity values exceeding 20,000 MET-min/week were inspected for potential outliers. Cases with clearly invalid or inconsistent activity patterns were excluded during data screening. The final IPAQ scores represented total habitual physical activity across daily life domains. The IPAQ-SF has demonstrated acceptable reliability and validity in diverse populations and cultural contexts, including established psychometric properties in Chinese samples (Chang and Li, 2025).

#### 2.3.2 Maslach burnout inventory-student survey (MBI-SS)

Academic burnout was measured using the Maslach Burnout Inventory-Student Survey (MBI-SS), a standardized instrument adapted from the original Maslach Burnout Inventory for human service professions to the academic context (Yavuz and Dogan, 2014). The MBI-SS comprises 15 items distributed across three core subscales: Emotional Exhaustion (5 items; e.g., “I feel emotionally drained by my studies”), Cynicism (4 items; e.g., “I have become more cynical about the potential usefulness of my studies”), and Reduced Academic Efficacy (6 items; e.g., “I can effectively solve the problems that arise in my studies” [reverse-scored]). All items are rated on a 7-point frequency scale ranging from 0 (Never) to 6 (Always). Subscale scores are calculated by summing the responses for items belonging to each dimension. A global composite score of academic burnout can also be derived, with higher scores on the Emotional Exhaustion and Cynicism subscales, and lower scores on the Academic Efficacy subscale (after reverse-coding), indicating a higher level of burnout. The Chinese version of the MBI-SS employed in this study has demonstrated sound psychometric properties, including good internal consistency, test–retest reliability, and convergent validity in previous research with Chinese student populations (Hernesniemi et al., 2017). In the present sample, the scale showed good internal consistency (Cronbach’s *α* = 0.89 at T1, and Cronbach’s *α* = 0.91 at T2).

#### 2.3.3 Self-efficacy

General self-efficacy was assessed using the 10-item General Self-Efficacy Scale (GSES) developed by Luszczynska et al. (2005). The scale is designed to measure an individual’s stable, optimistic belief in their ability to cope with a wide range of difficult demands and challenges in life. It includes items such as “I can always manage to solve difficult problems if I try hard enough,” and “I am confident that I could deal efficiently with unexpected events.” Responses to each item are recorded on a 4-point Likert scale, ranging from 1 (Not at all true) to 4 (Exactly true). A total summary score is calculated by summing the responses across all 10 items, yielding a possible range from 10 to 40, where higher scores indicate a stronger sense of general self-efficacy. The GSES is a widely used and internationally validated instrument. Its Chinese version has demonstrated strong psychometric properties, including high internal consistency, a stable one-dimensional factorial structure, and good convergent validity in numerous studies with Chinese populations (Schwarzer et al., 1997). In the present sample, the scale showed good internal consistency (Cronbach’s *α* = 0.86 at T1 and Cronbach’s *α* = 0.89 at T2).

#### 2.3.4 Professionalism

Professionalism was evaluated using the professionalism Scale for Medical Students (PAS), a 22-item self-report instrument developed by Klemenc-Ketis and Vrecko (2014) to measure multifaceted professional attitudes and behaviors in medical education. The scale is composed of three distinct dimensions: Empathy and Humanism (EH), assessed by 8 items (e.g., “I can put aside my biases while providing care to patients”); Professional Relationship and Development (PR-D), assessed by 6 items (e.g., “I actively seek feedback to improve my professional skills”); and Responsibility (R), assessed by 8 items (e.g., “I consistently complete my clinical tasks with thoroughness and diligence”). All items are positively worded and rated on a 5-point Likert scale ranging from 1 (Strongly Disagree) to 5 (Strongly Agree). A total score is calculated by summing all 22 item responses, yielding a possible range from 22 to 110. Subscale scores for each dimension are computed by summing the items within that dimension. Higher scores on both the subscales and the total score indicate more positive professional attitudes and greater perceived professionalism. Although professionalism is inherently multidimensional and ideally assessed using behavioral observations, peer evaluations, or supervisor ratings, self-report measures remain widely used in medical education research to assess professionalism-related attitudes, values, and self-perceptions. In the present study, the PAS was used to capture students’ perceived professionalism rather than objective professional competence or observed clinical performance. Accordingly, the findings should be interpreted as reflecting self-perceived professionalism-related dispositions within medical training contexts. The PAS has demonstrated good psychometric properties in previous validation studies with Chinese medical students, including acceptable internal consistency, test–retest reliability, and convergent validity (Yang et al., 2013). In the present sample, the scale also showed good internal consistency (Cronbach’s *α* = 0.91 at T1 and Cronbach’s *α* = 0.92 at T2).

#### 2.3.5 Longitudinal organization of variables

To improve methodological transparency, all primary variables were measured at both Time 1 (T1) and Time 2 (T2). In the hypothesized prospective indirect-effect model, T1 physical activity was specified as the distal predictor, whereas T2 academic burnout and T2 self-efficacy were specified as parallel mediators. T2 professionalism served as the outcome variable.

### 2.4 Data analysis

Data analysis was conducted using IBM SPSS Statistics (Version 27.0) and Mplus (Version 8.3). First, descriptive statistics (means, standard deviations, and frequencies) were calculated for all study variables. Internal consistency reliability for all scales was evaluated separately at T1 and T2 using Cronbach’s alpha coefficients. Pearson correlation analyses were then conducted to examine both synchronous and asynchronous associations among physical activity, academic burnout, self-efficacy, and perceived professionalism across the two measurement waves. In addition, partial correlations controlling for baseline levels of burnout, self-efficacy, and professionalism were calculated to preliminarily assess prospective associations among the study variables.

Physical activity was assessed using the International Physical Activity Questionnaire–Short Form (IPAQ-SF). Weekly MET-min/week scores were calculated according to the official IPAQ scoring protocol. Data-cleaning procedures followed established IPAQ recommendations. Reported activity durations exceeding 180 min per day within a single activity category were truncated to reduce the influence of implausible overreporting. Total physical activity scores exceeding 20,000 MET-min/week were additionally screened for potential outliers and inconsistent response patterns. Cases with clearly invalid responses were excluded during data screening. The final IPAQ score represented habitual total physical activity across daily life domains rather than structured exercise participation specifically.

Validated Chinese versions of all instruments were used without item modification. Cronbach’s *α* coefficients for the MBI-SS were 0.89 at T1 and 0.91 at T2. For the General Self-Efficacy Scale (GSES), α values were 0.86 at T1 and 0.89 at T2. The Professionalism Assessment Scale (PAS) demonstrated α coefficients of 0.91 at T1 and 0.92 at T2, indicating satisfactory internal consistency across both measurement waves.

Prior to testing the structural model, confirmatory factor analyses (CFA) were conducted to evaluate the adequacy of the latent measurement model. Academic burnout, self-efficacy, and perceived professionalism were modeled as latent constructs using parcel indicators. Parceling was considered appropriate because preliminary item-level CFAs demonstrated acceptable unidimensionality within each construct together with strong internal consistency reliability.

The item-to-construct balance approach was used to create parcels. This method distributes items with relatively higher and lower factor loadings evenly across parcels to improve indicator balance and reduce the risk of parcel-specific bias. For the 15-item MBI-SS, three parcels were created: Parcel 1 included items 1, 4, 7, 10, and 13; Parcel 2 included items 2, 5, 8, 11, and 14; and Parcel 3 included items 3, 6, 9, 12, and 15. For the 10-item GSES, three parcels were created: Parcel 1 included items 1, 4, 7, and 10; Parcel 2 included items 2, 5, and 8; and Parcel 3 included items 3, 6, and 9. For the 22-item PAS, three parcels were also created using the same balanced allocation principle. Parcel 1 included items 1, 4, 7, 10, 13, 16, 19, and 22; Parcel 2 included items 2, 5, 8, 11, 14, 17, and 20; and Parcel 3 included items 3, 6, 9, 12, 15, 18, and 21. The same parceling structure was applied consistently across T1 and T2.

Parceling was adopted to reduce model complexity, improve indicator reliability, and enhance parameter stability in the longitudinal SEM analyses. Standardized factor loadings, composite reliability (CR), and average variance extracted (AVE) were examined to evaluate convergent validity. Discriminant validity was assessed by examining latent factor correlations and comparing the square root of AVE values with inter-factor correlations.

Longitudinal measurement invariance across T1 and T2 was subsequently examined sequentially using configural, metric, and scalar invariance models. Following established recommendations, invariance was considered acceptable when decreases in Comparative Fit Index (ΔCFI) were smaller than 0.010 and increases in Root Mean Square Error of Approximation (ΔRMSEA) were smaller than 0.015 between nested models.

The primary analysis tested a two-wave prospective indirect-effect model using Structural Equation Modeling (SEM) with maximum likelihood estimation. Physical activity (IPAQ total MET-min/week) was treated as an observed variable because it represented a composite behavioral score derived from the IPAQ-SF. Autoregressive effects were controlled by including T1 burnout, T1 self-efficacy, and T1 perceived professionalism as predictors of their corresponding T2 constructs.

Because the mediators (burnout and self-efficacy) and the outcome variable (perceived professionalism) were measured concurrently at T2, the present design cannot establish full longitudinal mediation in a strict temporal sense. Therefore, the indirect paths were interpreted as prospective indirect associations that are consistent with the hypothesized theoretical framework rather than definitive causal mediation effects. Nevertheless, controlling for baseline levels of the mediators and outcome strengthened the temporal interpretation of the observed associations.

Model fit was evaluated using multiple indices, including the χ^2^/df ratio (<3 indicating acceptable fit, <2 indicating excellent fit), Comparative Fit Index (CFI 0.90 acceptable, 0.95 excellent), Tucker–Lewis Index (TLI 0.90 acceptable, 0.95 excellent), Root Mean Square Error of Approximation (RMSEA < 0.08 acceptable, <0.05 excellent), and Standardized Root Mean Square Residual (SRMR < 0.08).

The significance of the prospective indirect associations (i.e., T1 physical activity → T2 burnout → T2 perceived professionalism and T1 physical activity → T2 self-efficacy → T2 perceived professionalism) was evaluated using a bias-corrected bootstrap procedure with 5,000 resamples. Indirect effects were considered statistically significant when the 95% confidence interval (CI) did not include zero. The relative magnitude of the two indirect pathways was also compared. Alternative models, including a partially mediated model containing a direct path from T1 physical activity to T2 perceived professionalism, were additionally compared to identify the most parsimonious and best-fitting model. Statistical significance was set at *α* = 0.05 (two-tailed).

## 3 Results

### 3.1 Sample description

As shown in Table 1, based on the demographic data collected from the participants (*N* = 625), the following characteristics were observed: The sample consisted of 281 males (45.0%) and 344 females (55.0%). Participants’ ages ranged from 18 to 26 years, with a mean age of 22.4 years (SD = 1.5). Participants were distributed across different academic levels: 156 (25.0%) were in Bachelor Year 4, 156 (25.0%) in Bachelor Year 5, 157 (25.1%) in Master Year 2, and 156 (25.0%) in Master Year 3. Regarding parental education, 376 participants (60.2%) reported that their parents had completed high school education or below, 156 (25.0%) reported parental education at the bachelor’s degree level, and 93 (14.9%) reported parental education at the master’s degree level or above.

**Table 1:** Sociological demographic characteristics (*n* = 625).

Variable	Category	*n*	%
Gender	Male	281	45.0
	Female	344	55.0
Age (years)	Mean ± SD	22.4 ± 1.5	--
Year of Study	Bachelor Year 4	156	25.0
	Bachelor Year 5	156	25.0
	Master Year 2	157	25.1
	Master Year 3	156	25.0
Parental Education	High School or below	376	60.2
	Bachelor	156	25.0
	Master or above	93	14.8

### 3.2 Descriptive statistics and normality tests

Table 2 presents the descriptive statistics and normality test results for all study variables at T1 and T2. The mean scores of physical activity were 1456.82 (SD = 432.170) at T1 and 1500.50 (SD = 422.362) at T2. The mean burnout scores were 2.98 (SD = 0.72) at T1 and 2.96 (SD = 0.72) at T2. Self-efficacy showed mean scores of 2.50 (SD = 0.66) at T1 and 2.46 (SD = 0.70) at T2, whereas PAS scores were 3.01 (SD = 0.66) at T1 and 3.02 (SD = 0.69) at T2.

**Table 2:** Descriptive statistics and normality test results for all variables at T1 and T2.

Variables	Mean	SD	Skewness	Kurtosis	Shapiro–Wilk *W*	*p*
T1 IPAQ	1456.820	432.170	0.024	−0.042	0.998	0.742
T2 IPAQ	1500.500	422.362	0.037	−0.081	0.997	0.418
T1 Burnout	2.983	0.717	0.112	−0.231	0.997	0.566
T2 Burnout	2.964	0.720	0.095	−0.248	0.997	0.642
T1 Self-Efficacy	2.500	0.656	−0.284	−0.114	0.990	0.001
T2 Self-Efficacy	2.463	0.697	−0.337	−0.076	0.986	< 0.001
T1 PAS	3.009	0.663	0.071	−0.301	0.997	0.515
T2 PAS	3.022	0.694	0.084	−0.287	0.996	0.213

The skewness and kurtosis values for all variables ranged between −1 and +1, indicating acceptable univariate normality. In addition, the Shapiro–Wilk statistics were generally acceptable given the relatively large sample size, supporting the suitability of subsequent parametric analyses and structural equation modeling.

### 3.3 Analysis of correlations between variables across time points

**Figure 1:**
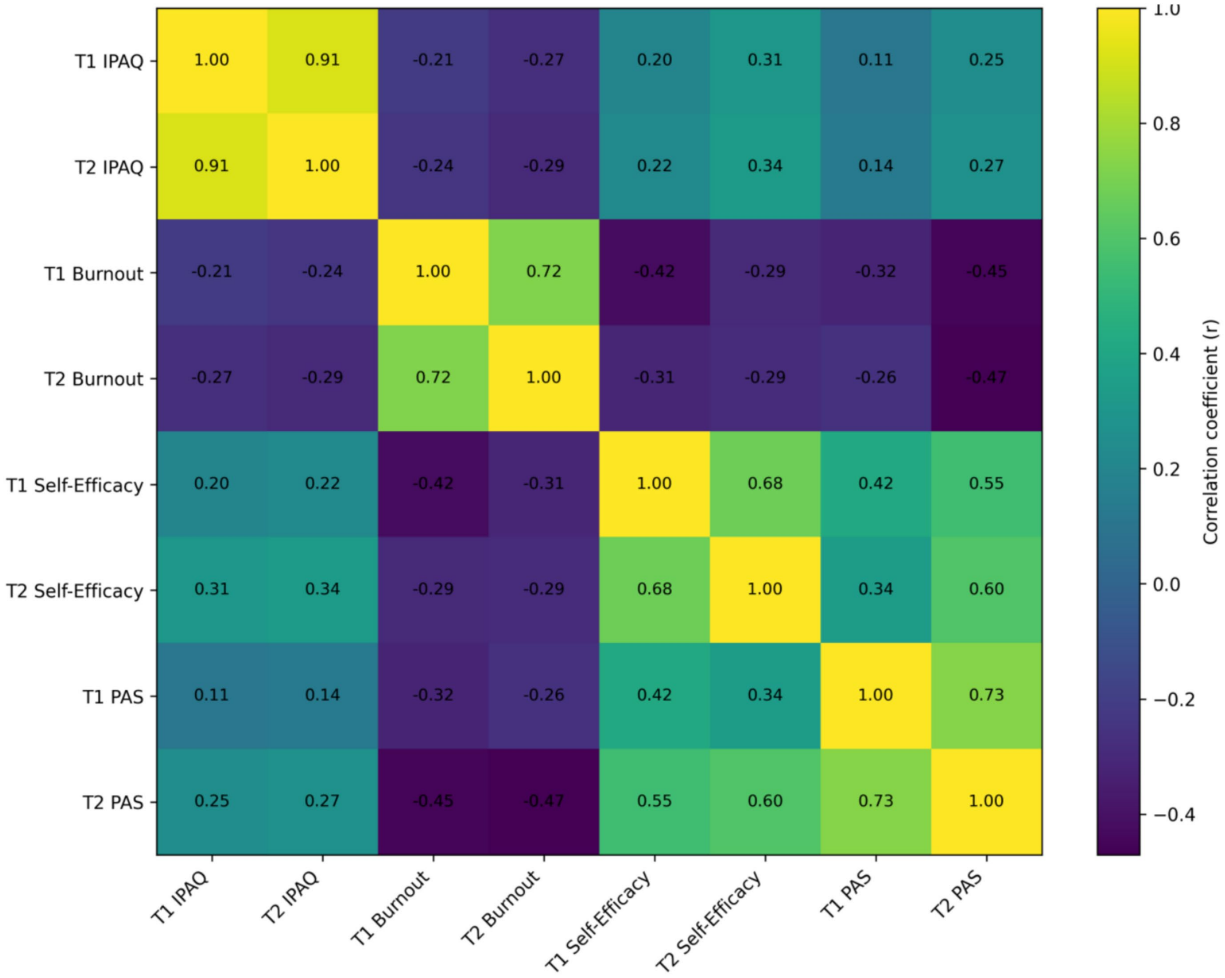
Correlation heatmap of all variables at T1 and T2.

Table 3 and Figure 1 presents the synchronous and asynchronous correlations among all study variables across the two measurement waves. Significant positive autoregressive correlations were found for physical activity (*r* = 0.914, *p* < 0.001), burnout (*r* = 0.724, *p* < 0.001), self-efficacy (*r* = 0.676, *p* < 0.001), and PAS (*r* = 0.731, *p* < 0.001), indicating good temporal stability over the 6-month interval.

**Table 3:** Synchronous and asynchronous correlations among study variables at T1 and T2.

Variables	1	2	3	4	5	6	7	8
1. T1 IPAQ	—							
2. T2 IPAQ	0.914***	—						
3. T1 Burnout	−0.214***	−0.236***	—					
4. T2 Burnout	−0.271***	−0.295***	0.724***	—				
5. T1 Self-Efficacy	0.201***	0.223***	−0.421***	−0.313***	—			
6. T2 Self-Efficacy	0.309***	0.336***	−0.295***	−0.286***	0.676***	—		
7. T1 PAS	0.112**	0.138***	−0.316***	−0.258***	0.424***	0.342***	—	
8. T2 PAS	0.248***	0.271***	−0.453***	−0.471***	0.548***	0.600***	0.731***	—

**p < 0.01, ***p < 0.001.

At both time points, physical activity was negatively correlated with burnout and positively correlated with self-efficacy and PAS. Specifically, T1 physical activity was negatively associated with T2 burnout (*r* = −0.271, *p* < 0.001) and positively associated with T2 self-efficacy (*r* = 0.309, *p* < 0.001) and T2 PAS (*r* = 0.248, *p* < 0.001). In addition, burnout was negatively correlated with self-efficacy and PAS across both waves, whereas self-efficacy showed significant positive correlations with PAS. These findings preliminarily supported the hypothesized longitudinal associations among the study variables.

### 3.4 Test for common methods bias

To examine for potential common method bias, this study employed Harman’s one-factor test. Unrotated principal component analysis was performed on all scale items (15 items for academic burnout, 10 items for self-efficacy, and 22 items for professionalism, totaling 47 items). The results of Table 4 showed that the first factor explained 24.55% of the variance, below the 40% critical value. The first five factors cumulatively explained 54.59% of the variance, and seven factors had eigenvalues greater than 1 (Kaiser’s criterion). These results indicate that the data in this study do not suffer from serious common method bias.

**Table 4:** Harman’s one-way factorial test results.

Factor	Eigen values	Explained variance (%)	Cumulative explained variance (%)
Factor 1	11.537	24.55	24.55
Factor 2	5.892	12.54	37.09
Factor 3	3.214	6.84	43.93
Factor 4	2.876	6.12	50.05
Factor 5	2.134	4.54	54.59
Other factor	21.331	45.41	100.00
Total	47.000	100.00	100.00

### 3.5 Measurement model evaluation

Prior to testing the structural model, a confirmatory factor analysis (CFA) was conducted to evaluate the adequacy of the latent measurement model. The measurement model demonstrated satisfactory fit to the data: *χ*^2^(120) = 248.37, *p* < 0.001, *χ*^2^/df = 2.07, CFI = 0.964, TLI = 0.956, RMSEA = 0.041, and SRMR = 0.036.

All parcel indicators loaded significantly onto their intended latent constructs, with standardized factor loadings ranging from 0.71 to 0.89 (all *p* < 0.001). Composite reliability (CR) values ranged from 0.87 to 0.94, and average variance extracted (AVE) values ranged from 0.62 to 0.78, supporting satisfactory convergent validity. In addition, the square root of each construct’s AVE exceeded its correlations with other latent constructs, indicating acceptable discriminant validity.

These findings supported the adequacy of the latent measurement structure and justified proceeding to the longitudinal invariance and structural analyses.

### 3.6 Measurement invariance

Following establishment of the baseline measurement model, longitudinal measurement invariance across T1 and T2 was examined sequentially using configural, metric, and scalar invariance models (Table 5). The configural invariance model demonstrated excellent fit to the data (*χ*^2^ = 120.36, df = 120, CFI = 0.999, TLI = 0.999, RMSEA = 0.002), supporting a consistent factor structure across time. Constraining factor loadings to equality across T1 and T2 produced minimal changes in model fit (ΔCFI = 0.000, ΔRMSEA = 0.007), supporting metric invariance. Further constraining item intercepts also resulted in acceptable changes in fit indices (ΔCFI = 0.001, ΔRMSEA = 0.002), indicating scalar invariance across the two measurement waves. These findings suggest that burnout, self-efficacy, and professionalism were measured equivalently across time, thereby supporting the validity of the longitudinal SEM analyses.

**Table 5:** Longitudinal measurement invariance across T1 and T2.

Model	*χ* ^2^	df	CFI	TLI	RMSEA	ΔCFI	ΔRMSEA
Configural invariance	120.36	120	0.999	0.999	0.002	–	–
Metric invariance	132.22	126	0.999	0.999	0.009	0.000	0.007
Scalar invariance	145.81	132	0.998	0.998	0.011	0.001	0.002

### 3.7 Structural equation modeling: testing the prospective indirect-effect model

**Figure 2:**
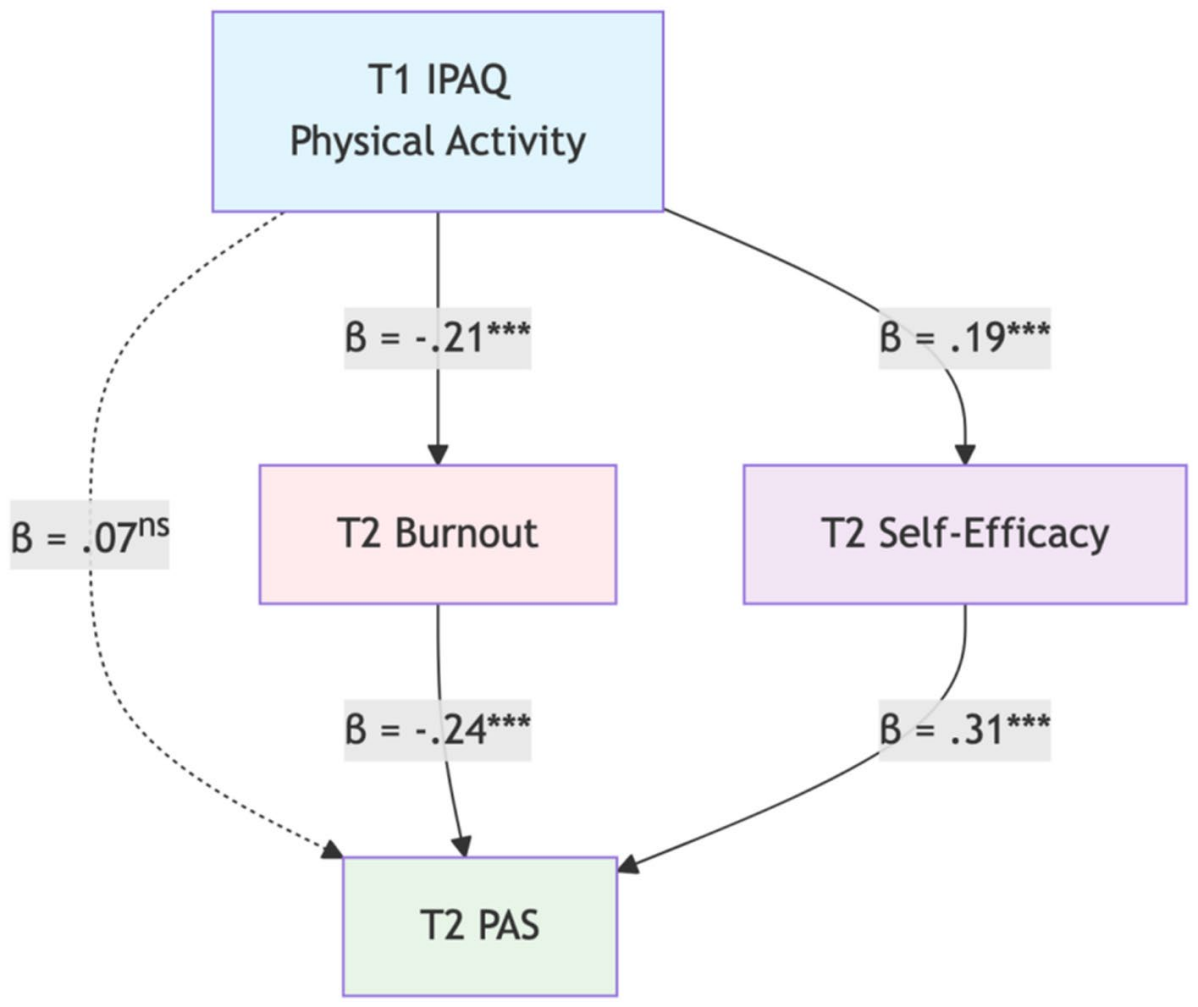
Standardized path coefficients for the final dual-mediation model. *** means that the path coefficient is significant.

After establishing the adequacy of the measurement model and longitudinal measurement invariance, the hypothesized prospective indirect-effect structural model was tested using SEM (Table 6 and Figure 2). The model demonstrated an excellent fit to the data: *χ*^2^(142) = 285.67, *p* < 0.001, *χ*^2^/df = 2.01, CFI = 0.968, TLI = 0.961, RMSEA = 0.040 (90% CI: 0.033, 0.047), SRMR = 0.038. All fit indices met the recommended criteria for good model fit. Direct effects. Time 1 physical activity (IPAQ) negatively predicted Time 2 academic burnout (*β* = −0.21, SE = 0.04, *p* < 0.001) and positively predicted Time 2 self-efficacy (*β* = 0.19, SE = 0.04, *p* < 0.001). Time 2 academic burnout negatively predicted Time 2 professionalism (*β* = −0.24, SE = 0.04, *p* < 0.001), while Time 2 self-efficacy positively predicted it (*β* = 0.31, SE = 0.04, *p* < 0.001). The direct path from Time 1 physical activity to Time 2 professionalism was non-significant (*β* = 0.07, SE = 0.04, *p* = 0.102). Autoregressive controls. Significant autoregressive effects were observed for burnout (*β* = 0.58, SE = 0.03, *p* < 0.001), self-efficacy (*β* = 0.56, SE = 0.03, *p* < 0.001), and professionalism (*β* = 0.52, SE = 0.03, *p* < 0.001).

**Table 6:** Standardized direct, indirect, and total effects in the structural model (*N* = 625).

Path	*β*	SE	*p*	95% CI
Direct Effects				
T1 IPAQ → T2 Burnout	−0.21	0.04	<0.001	[−0.29, −0.13]
T1 IPAQ → T2 Self-Efficacy	0.19	0.04	<0.001	[0.11, 0.27]
T2 Burnout → T2 PAS	−0.24	0.04	<0.001	[−0.32, −0.16]
T2 Self-Efficacy → T2 PAS	0.31	0.04	<0.001	[0.23, 0.39]
T1 IPAQ → T2 PAS (direct)	0.07	0.04	0.102	[−0.01, 0.15]
Autoregressive controls				
T1 Burnout → T2 Burnout	0.58	0.03	<0.001	[0.52, 0.64]
T1 Self-Efficacy → T2 Self-Efficacy	0.56	0.03	<0.001	[0.50, 0.62]
T1 PAS → T2 PAS	0.52	0.03	<0.001	[0.46, 0.58]
Indirect effects (bootstrapped)				
T1 IPAQ → T2 Burnout → T2 PAS	0.05	0.01	<0.001	[0.03, 0.08]
T1 IPAQ → T2 Self-Efficacy → T2 PAS	0.06	0.01	<0.001	[0.04, 0.09]
Total Indirect Effect	0.11	0.02	<0.001	[0.07, 0.15]
Total Effect (Direct + Indirect)	0.18	0.04	<0.001	[0.10, 0.26]

β, standardized path coefficient; SE, standard error; CI, confidence interval. All mediator and outcome models control for gender, age, and year of study (coefficients not shown for brevity).

Indirect and total effects. Bootstrapping analyses (5,000 resamples) indicated prospective significant indirect effects of physical activity on professionalism via both burnout (*β* = 0.05, SE = 0.01, *p* < 0.001) and self-efficacy (*β* = 0.06, SE = 0.01, *p* < 0.001). The total indirect effect was *β* = 0.11 (SE = 0.02, *p* < 0.001). The total effect (direct + indirect) was *β* = 0.18 (SE = 0.04, *p* < 0.001).

### 3.8 Comparison of mediation effects in parallel mediation models

To examine the relative strength of the two mediation pathways, we conducted a mediation effect difference test (see Table 7). Bootstrap analysis (5,000 resamples) revealed that the indirect effect through self-efficacy (*β* = 0.06, 95% CI [0.04, 0.09]) was slightly larger than that through burnout (*β* = 0.05, 95% CI [0.03, 0.08]), but this difference did not reach statistical significance (difference *β* = 0.01, 95% CI [−0.02, 0.04], *p* = 0.48).

**Table 7:** Decomposition and comparison of mediation effects in the parallel mediation model (*N* = 625).

Path	Type of effect	*β*	SE	*p*	95% CI
Direct effects					
T1 IPAQ → T2 PAS	Direct Effect	0.07	0.04	0.102	[−0.01, 0.15]
Indirect effects					
T1 IPAQ → T2 Burnout → T2 PAS	Indirect Effect 1	0.05	0.01	<0.001	[0.03, 0.08]
T1 IPAQ → T2 Self-Efficacy → T2 PAS	Indirect Effect 2	0.06	0.01	<0.001	[0.04, 0.09]
Comparison of mediation effects					
Self-Efficacy path – Burnout path	Effect Difference	0.01	0.01	0.48	[−0.02, 0.04]
Total effect					
T1 IPAQ → T2 PAS	Total Effect	0.18	0.04	<0.001	[0.10, 0.26]

β, standardized path coefficient; SE, standard error; CI, confidence interval. All models control for gender, age, year of study, and baseline levels of mediators and outcome variable. Confidence intervals for indirect effects and effect differences were estimated using bootstrapping with 5,000 resamples.

## 4 Discussion

This study examined whether physical activity was prospectively associated with perceived professionalism among medical students through academic burnout and self-efficacy. Given that the study used a two-wave observational design and that the mediators and outcome were assessed concurrently at T2, the findings should not be interpreted as evidence of causal or full longitudinal mediation. Rather, the results indicate a pattern of prospective indirect associations that is consistent with the proposed theoretical framework.

### 4.1 Summary and interpretation of key findings

Our findings corroborate the central proposition that regular physical activity serves as a significant distal antecedent to professionalism, but its related to is entirely channeled through intermediate psychological states rather than being direct. The excellent fit of the structural model and the significance of both specified indirect paths strongly support H5. Specifically, higher levels of physical activity at baseline predicted lower subsequent academic burnout (supporting H1) and higher subsequent general self-efficacy (supporting H2). In turn, these T2 psychological states were potent predictors of T2 professionalism: burnout negatively (supporting H3) and self-efficacy positively (supporting H4). The non-significant direct path from T1 exercise to T2 professionalism, coupled with significant total and indirect effects, confirms full parallel mediation. This pattern suggests that exercise does not impact professional attitudes and perceived professionalism in a vacuum (Rodgers et al., 2014) instead, its benefits are contingent upon first improving the student’s psychological resource landscape by conserving resources drained by chronic academic stress and by building a stronger sense of personal agency.

The exploratory comparison of the two mediation pathways (H6) yielded a critical nuance. Although the point estimate for the self-efficacy path was slightly larger (*β* = 0.06) than the burnout path (*β* = 0.05), this difference was not statistically significant. This indicates that, within our model, both pathways contribute in a relatively balanced and statistically equivalent manner to the professionalism relationship. Therefore, it is not accurate to characterize the mechanism as being primarily protective or primarily enabling; rather, physical activity appears to operate through a dual-process system that concurrently mitigates a key risk factor (burnout) and amplifies a key personal resource (self-efficacy). This balanced synergy underscores the multifaceted psychological value of exercise in a high-demand training environment.

### 4.2 Theoretical integration and implications

The results offer strong empirical validation for the integration of Conservation of Resources (COR) Theory and Social Cognitive Theory (SCT) in understanding how a health behavior translates into professionalism-related development. From a COR perspective, medical training represents a prolonged threat of resource loss, with burnout as its hallmark (Gaston-Hawkins et al., 2020). Our finding that exercise predicts reduced burnout longitudinally aligns with the concept of “resource investment.” Exercise, though an initial expenditure of energy and time, functions as a strategic investment that builds physiological and psychological resilience, thereby preventing the severe resource depletion spiral characteristic of burnout (Kuvaja-Köllner et al., 2023). By mitigating exhaustion and cynicism, exercise helps preserve the emotional and cognitive resources necessary for engaging in the relational, reflective, and ethically demanding tasks that constitute professionalism.

Simultaneously, the significant path through self-efficacy powerfully illustrates the agentic dimension of SCT. The mastery experiences inherent in setting and achieving exercise goals whether completing a run, increasing weights, or improving technique provide concrete, unambiguous evidence of personal capability (Min, 2023). These experiences in the physical domain likely foster a generalized sense of self-efficacy, which then transfers to the academic and clinical domains. An individual who believes they can overcome the challenge of a strenuous workout may also develop greater confidence in their ability to master complex medical knowledge or navigate a difficult patient conversation. This enhanced belief in one’s own efficacy is directly conducive to professional behaviors such as proactive learning, perseverance in the face of setbacks, and confident communication all core components measured by the PAS.

The fact that both pathways were significant and equally potent enriches our theoretical understanding. It suggests that interventions aimed at fostering professionalism cannot focus solely on stress reduction (the deficit model) or solely on confidence building (the strength-based model). A comprehensive approach should recognize that students need both: protection from the erosive effects of their environment and active cultivation of the internal beliefs that drive growth and engagement. Physical activity, as our model demonstrates, may uniquely serve this dual purpose.

### 4.3 Contextualization within existing literature

Our findings extend the existing literature in several important ways. First, while prior studies have established cross-sectional links between physical activity and lower burnout or higher well-being in students (Chang and Li, 2025), our longitudinal design with autoregressive controls provides stronger evidence for the hypothesized temporal and potentially is associated with sequence. The significant prediction of T2 outcomes after controlling for their robust T1 stability (autoregressive paths ranging from 0.52 to 0.65) underscores that exercise is associated with change in these psychological constructs over time.

Second, most research on exercise and student outcomes terminates at general mental health or academic performance (Eisenberg et al., 2009; Suldo et al., 2014). By linking exercise specifically to professionalism a multidimensional construct central to medical education, we bridge a significant gap. We move beyond asking if exercise helps students “feel better” or “score higher” to demonstrate how it might help them “become better” future physicians. This aligns with and expands the emerging discourse on “lifestyle medicine for the medical trainee,” positioning personal health practices as foundational to professional identity formation (Razavi et al., 2020).

Third, our parallel mediation model addresses a fragmentation in the literature. Previous studies often examine burnout or self-efficacy in isolation. By modeling them simultaneously, we could assess their unique contributions and reveal their complementary, non-competitive roles. The lack of a significant difference between the two indirect effects suggests that the literature’s occasional emphasis on one pathway over the other may be more a function of research focus than a true reflection of their relative importance in the naturalistic process.

### 4.4 Practical implications for medical education

The results carry concrete implications for curriculum design and student support in medical schools. Administrators and educators should actively promote and facilitate regular physical activity as a legitimate and valuable component of professionalism-related development, not merely a leisure activity. This could involve: Structural Support, Integrating protected time for physical activity within busy academic schedules, providing on-campus fitness facilities at subsidized rates, and offering a variety of structured exercise programs (e.g., group sports, yoga, mindfulness-based movement). Educational Integration, Incorporating discussions about physician self-care and resilience into the formal curriculum, using evidence like this study to frame exercise as a strategic tool for sustaining performance and professionalism, akin to training in clinical skills. Early Intervention, Encouraging exercise habits from the early years of medical school, as our model suggests its benefits accumulate through psychological mechanisms over time. Peer-led exercise groups or mentoring programs could be effective. Holistic Advising, Academic advisors and wellness counselors should routinely assess physical activity levels alongside academic performance and psychological distress, providing guidance and resources to students showing low engagement.

### 4.5 Limitations and future research directions

Several limitations of this study should be acknowledged. First, while longitudinal, the design remains observational. Although we controlled for baseline levels, unmeasured confounding variables (e.g., personality traits like conscientiousness, concurrent life events) could related to both exercise adherence and the psychological mediators. Future research would benefit from randomized controlled trials (RCTs) that manipulate exercise engagement to establish is associated with efficacy.

Second, all key variables are self-reported. Especially physical activity was measured via self-report (IPAQ-SF), which is subject to recall and social desirability biases. Objective measures like accelerometers would provide more precise data on activity intensity, frequency, and duration, allowing for dose–response analyses (e.g., what “dose” of exercise is most beneficial?).

Two methodological limitation should be considered when interpreting the indirect effects. First, common-method bias is not adequately ruled out by Harman’s test alone. Second, although the study adopted a two-wave prospective design and controlled for baseline levels of burnout, self-efficacy, and professionalism, the mediators and outcome were assessed concurrently at T2. As a result, the temporal ordering between the mediators and professionalism cannot be fully determined. Accordingly, the indirect effects should be interpreted as prospective indirect associations that are consistent with the proposed theoretical framework rather than conclusive evidence of longitudinal mediation. Future studies using at least three measurement waves would provide a more rigorous examination of temporal mediation processes.

An additional limitation is the exclusion of students with physical conditions restricting physical activity participation. Although this criterion improved the interpretability of IPAQ-SF assessments, it may have introduced selection bias and limited the generalizability of the findings to relatively healthy medical student populations.

Finally, the study was conducted within a specific cultural context (Chinese medical universities). The generalizability of the findings to other educational systems and cultures needs to be verified. Cross-cultural comparisons could reveal how institutional and cultural factors moderate the observed pathways.

## 5 Conclusion

In conclusion, this two-wave prospective study found that physical activity was indirectly associated with self-perceived professionalism among medical students through lower academic burnout and higher self-efficacy. These findings suggest that habitual physical activity may be linked to psychological conditions relevant to professionalism-related attitudes and engagement during medical training. However, because professionalism was assessed using a self-report measure, the findings should not be interpreted as evidence of objective professional competence or actual clinical performance. Future studies incorporating behavioral and multi-source assessments are needed to further clarify these relationships.

## Data Availability

The original contributions presented in the study are included in the article/supplementary material, further inquiries can be directed to the corresponding author.
